# Axonal Transport Defect in Gigaxonin Deficiency Rescued by Tubastatin A

**DOI:** 10.1007/s13311-023-01393-1

**Published:** 2023-06-02

**Authors:** Banshi Nath, Daniel Phaneuf, Jean-Pierre Julien

**Affiliations:** 1 CERVO Brain Research Centre, 2601, de La Canardière, Québec City, Québec G1J2G3 Canada; 2 Department of Psychiatry and Neuroscience, Université Laval, Québec City, Québec Canada

**Keywords:** GAN Giant axonal neuropathy, Gan Gigaxonin, Neuropathy, IF Intermediate filament, NF Neurofilament, DRG Dorsal root ganglia, Nefl Neurofilament light chain, Prph Peripherin

## Abstract

Giant axonal neuropathy (GAN) is a disease caused by a deficiency of gigaxonin, a mediator of the degradation of intermediate filament (IF) proteins. A lack of gigaxonin alters the turnover of IF proteins, provoking accumulation and disorganization of neurofilaments (NFs) in neurons, a hallmark of the disease. However, the effects of IF disorganization on neuronal function remain unknown. Here, we report that cultured embryonic dorsal root ganglia (DRG) neurons derived from *Gan*^*−/−*^ mice exhibit accumulations of IF proteins and defects in fast axonal transport of organelles. Kymographs generated by time-lapse microscopy revealed substantial reduction of anterograde movements of mitochondria and lysosomes in axons of *Gan*^*−/−*^ DRG neurons. Treatment of *Gan*^*−/−*^ DRG neurons with Tubastatin A (TubA) increased the levels of acetylated tubulin and it restored the normal axonal transport of these organelles. Furthermore, we tested the effects of TubA in a new mouse model of GAN consisting of *Gan*^*−/−*^ mice with overexpression of peripherin (Prph) transgene. Treatment of 12-month-old *Gan*^*−/−*^;TgPer mice with TubA led to a slight amelioration of motor function, especially a significant improvement of gait performance as measured by footprint analyses. Moreover, TubA treatment reduced the abnormal accumulations of Prph and NF proteins in spinal neurons and it boosted the levels of Prph transported into peripheral nerve axons. These results suggest that drug inhibitors of histone deacetylase aiming to enhance axonal transport should be considered as a potential treatment for GAN disease.

**Supplementary Information:**

The online version contains supplementary material available at 10.1007/s13311-023-01393-1.

## Introduction

Giant axonal neuropathy GAN is an autosomal recessive neurodegenerative disorder characterized by the deterioration of the central (CNS) and peripheral nervous system (PNS) [1–3]. GAN is caused by mutations in the gene encoding gigaxonin, a protein of the BTB/Kelch family which is a mediator of degradation of intermediate filament (IF) proteins [3]. A deficiency in gigaxonin provokes accumulations and disorganization of neurofilaments (NFs) in the CNS and PNS, a hallmark of GAN disease [1, 2, 4, 5].

The formation of abnormal NF accumulations is sometimes associated with defective axonal transport of organelles. In Charcot-Marie-Tooth (CMT) type 2E neuropathy caused by *NEFL* mutations, there is evidence that accumulations of NFs can sequester mitochondria and interfere with their axonal transport [6, 7]. Cultured neurons with disorganized IFs, due to overexpression of peripherin (Prph) in the context of Nefl depletion, exhibited aberrant axonal transport of mitochondria with increased proportion of retrograde mitochondrial movements [8]. Moreover, in cultured dorsal root ganglion (DRG) neurons from *Gan*^*−/−*^ mice, mitochondria were found to be enriched at sites of NF accumulations and their movements were reduced [9].

Axonal transport of organelles is crucial for neuronal function and survival. The transport of organelles along microtubules is carried out by kinesin and dynein molecular motors which drive anterograde and retrograde movements, respectively [10]. The kinesin head, which remains bound to microtubules, hydrolyses ATP and moves the cargo towards the microtubule plus end. The docking of kinesin to microtubules is promoted by tubulin acetylation [11–13]. Tubulin acetylation is a reversible post-translational modification, and it is impeded by histone deacetylase 6 (HDAC6) activity [14–17]. Impairment of axonal transport has emerged as a common feature in several neurodegenerative disorders, including Alzheimer’s disease [18], Parkinson’s disease [19], Huntington’s disease [15], amyotrophic lateral sclerosis (ALS) [20] and Charcot-Marie-Tooth (CMT) [6, 21].

Here, we report that a gigaxonin deficiency in cultured DRG neurons from *Gan*^*−/−*^ mouse embryos causes IF accumulations and also axonal transport defects of organelles. Kymographs generated by time-lapse microscopy revealed that the anterograde movements of mitochondria and lysosomes were substantially reduced in axons of *Gan*^*−/−*^ DRG neurons. Treatment with Tubastatin A (TubA), an inhibitor of histone deacetylase 6 enzyme (HDAC6) previously shown to confer protection in mutant HSPB1-induced neuropathy [22], increased the levels of acetylated tubulin and restored the normal axonal transport of these organelles in *Gan*^*−/−*^DRG neurons. Furthermore, we tested the effects of TubA in a new mouse model of GAN with overt motor deficits [23], which was obtained by overexpressing a Prph transgene [24] in *Gan*^*−/−*^ mice [4]. Treatment of *Gan*^*−/−*^;TgPer mice with TubA for a period of 8 weeks, starting at 12 months of age, led to a significant improvement in gait performance determined by footprint analyses. Immunofluorescence microscopy of the spinal cord revealed that TubA treatment reduced the abnormal neuronal swellings due to IF accumulations. Consistent with microscopy data, western blotting after gel electrophoresis of proteins from the sciatic nerve showed that TubA boosted the levels of acetylated tubulin and the levels of Prph transported into peripheral nerve axons. These results suggest that drug inhibitors of HDAC6 aiming to rescue axonal transport defects should be considered as a potential treatment for GAN disease.

## Material and Methods

### Primary Culture

**Fig. 1:**
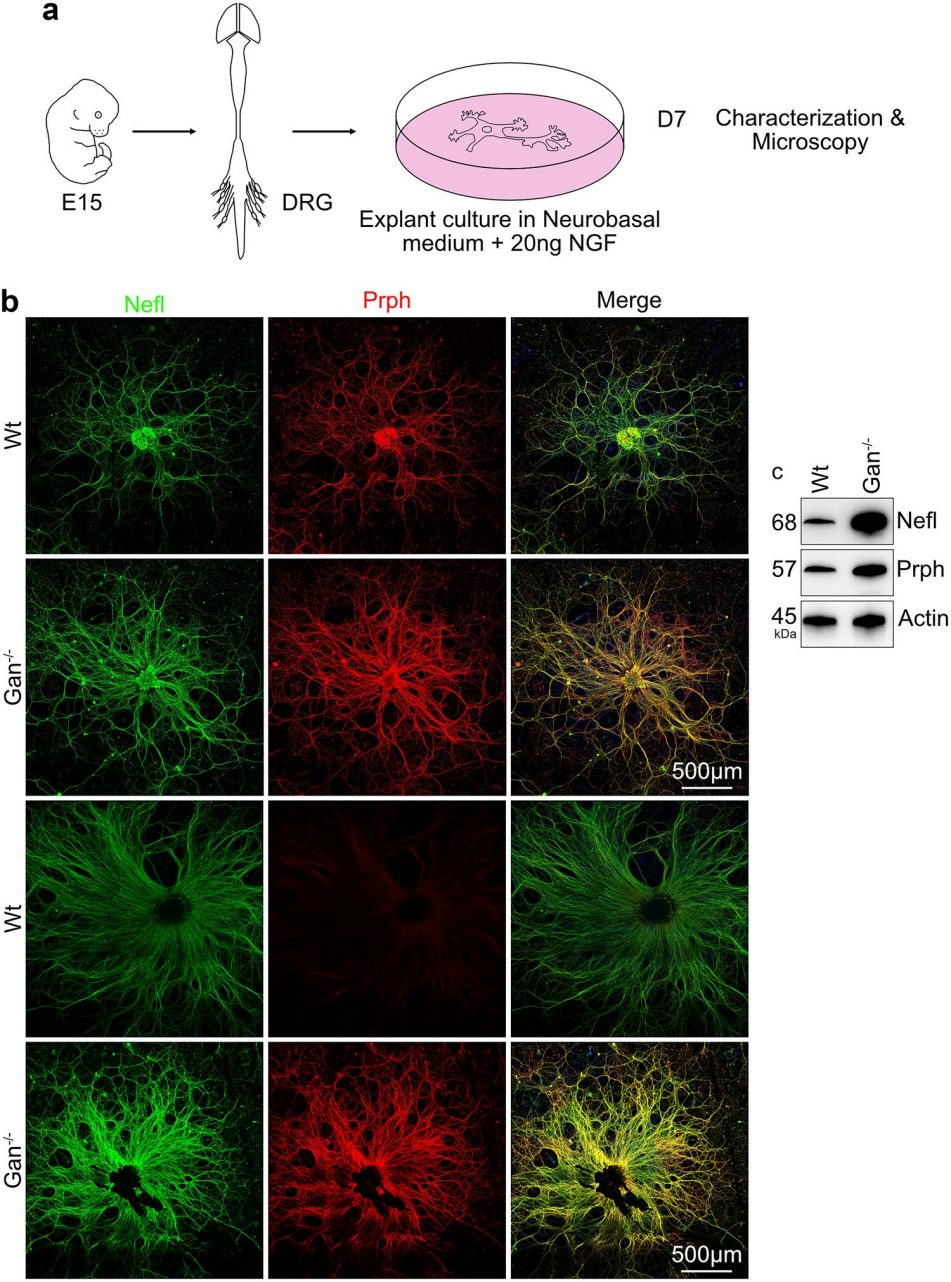
Increased levels of IF proteins in cultured *Gan*^*−/−*^ DRG neurons. Schematic representation shows the DRG culture from *Wt* and *Gan*^*−/−*^ embryos (**a**). Image panel (**b**) shows Nefl and Prph immunostaining in *Wt* and *Gan*^*−/−*^ DRG neurons. The *Gan*^*−/−*^ DRG neurons exhibit prominent immunostaining of Nefl and Prph, n = 3 independent DRG explant cultures. It is noteworthy that the Prph signal of *Wt* cultured DRG neurons varied due to the different embryonic culture groups (**b**). Original magnification 25x. Scale bar 500 μm. Western blots (**c**) reveal increased levels of Nefl and Prph in *Gan*^*−/−*^ DRG neurons. Scale bar 100 µm. Original magnification 25x. The colors green, red, and blue represent Nefl, Prph, and DAPI

*Gan*^*−/−*^ mice were produced as described previously [4]. Intraperitoneal (IP) injection of Ketamine (10 mg/ml), Xylene (1 mg/ml) were administered into the pregnant *Wt* and *Gan*^*−/−*^ female prior to sacrifice. DRG explants were prepared at embryonic days 15 (E15) (Schematic representation Fig. 1a. Briefly, the embryos were collected and placed in Hibernate A medium (Brain Bits) and dissected in neurobasal medium (Stem Cell Tech, USA) on ice. DRGs were collected from embryos and stored in Neurobasal medium. Further, the DRGs were transferred into a fresh neurobasal medium and centrifuged at 300 × g for 5 min. At least, 3 to 4 DRGs were placed on a freshly poly-D-lysine (Sigma) coated glass bottom culture dish (MatTeck Corp. USA) or glass chamber slides (ThermoFisher). DRG explants were allowed to attach on surface for 1 h before adding fresh neurobasal medium supplemented with B-27 supplement (Gibco), glutamine (Gibco), penicillin/streptomycin (Gibco), and 15 ng/ml nerve growth factor (NGF 2.5 s, Gibco). Half medium was replaced at every alternate day. Microscopy was performed at DIV7. HEK293 cells were cultured in Dulbecco’s Modified Eagle Medium (Gibco) containing 10% fetal bovine serum (Gibco) and penicillin/streptomycin. 1 µM/ml TubastatinA hydrochloride (MedChem Express) overnight treatment was given to DRG neurons and HEK293 cells in the appropriate medium. Equal volume of DMSO (Sigma) was used for vehicle treatment.

### Immunostaining

At DIV7, the DRG explants were fixed in -20 °C cold acetone for 10 min and blocked for 2 h in PBST containing 4% bovine serum albumin (Biobasic) and 0.25% Triton X-100. For microscopy of the spinal cord, mice were anesthetized by intraperitoneal injection of Ketamine (10 mg/ml), Xylazine (1 mg/ml) prior to sacrifice. Transcardial perfusion was performed with ice-cold PBS followed by 4% PFA. Spinal cord samples were collected and stored in 4% PFA overnight followed by 30% sucrose storage at 4 ^0^C. Transverse sections of 25 µm thick were cut using sliding VT 1200S microtome (Leica Microtome). Spinal cord lumbar region sections were mounted on a glass slide and allowed to dry overnight. Sections were washed 3 × with PBS further blocking was performed by 10% goat serum (Invitrogen) in PBST for 2 h to reduce nonspecific antibody binding. Immunostaining was performed using primary anti neurofilament light chain mouse monoclonal (Nefl, Sigma; 1:1000), anti NeuN rabbit polyclonal (Cell Signaling; 1:1000), anti peripherin rabbit polyclonal (Prph, Abcam; 1:1000), and anti NeuN mouse monoclonal (Millipore; 1:1000) antibody in PBST. Primary antibodies were incubated overnight and washed in PBST for 3 × for 5 min each at room temperature. Alexa Fluor 488 goat anti-mouse IgG (ThermoFisher) and Alexa Fluor 594 goat anti-rabbit IgG (ThermoFisher) antibodies were incubated for 2 h for secondary antibody incubation followed by washing in PBST. DAPI (Invitrogen; 1:10000) incubation was given for 1 min to stain nuclei. After 3 washes in PBS, slides were mounted with Fluoromount-G^®^ (Southern Biotech). DRG neurons and spinal cord sections were visualized using NikonA1 confocal microscope at 25 × objective.

### Western Blotting

DRG neurons and HEK293 cells were homogenized in 1% SDS buffer. The spinal cord and sciatic nerve from mice were collected as explained above. Briefly, mice were anesthetized by intraperitoneal injection of Ketamine (10 mg/ml), Xylazine (1 mg/ml) prior to sacrifice. Transcardial perfusion was performed with ice-cold PBS followed by tissue collection and stored at -80 °C. Further, the total protein extract was prepared in 1% hot SDS buffer and centrifuged at 12000 × g for 20 min at room temperature. Supernatants of cell lysates were collected, and samples were prepared in 1X sample buffer. SDS polyacrylamide gel was run and transferred on PVDF membrane. Immunoblot was blocked in 4% BSA followed by overnight primary antibody incubation of anti neurofilament heavy chain mouse monoclonal (Nefh, Abcam; 1:1000), anti neurofilament medium chain mouse monoclonal (Nefm, Sigma; 1:1000), anti α-internexin mouse monoclonal (Sigma; 1:1000), anti Nefl mouse monoclonal (1:1000), anti Prph rabbit polyclonal (1:1000), anti NeuN rabbit polyclonal (1:500), anti acetylated tubulin mouse monoclonal (Sigma; 1:1000), anti α-tubulin rabbit polyclonal (Abcam; 1:1000), and anti actin rabbit monoclonal (Cell Signaling; 1:1000). The next day, blots were washed in PBST (0.1% Tween-20) and suitable secondary antibodies incubation of anti mouse HRP (Jackson ImmunoResearch Lab) or anti rabbit HRP (Jackson ImmunoResearch Lab) in 1:5000 dilution was given for 1 h followed by washing in PBST. Immunoblots were developed and acquired at appropriate exposure in Bio-Rad chemiluminescent developer (BioRad) with ECL reagent (BioRad). Immunoblot was analyzed using ImageJ.

### Microscopy

Live cell imaging of mitochondria and lysosomes was performed at DIV7. To visualize mitochondria, DRG explant neurons were incubated with 100 nM MitoTracker™ Green FM (ThermoFisher) for 1 min followed by neurobasal medium incubation for 1 h. Similarly, for lysosomes, DRG neurons were labelled with 100 nM LysoTracker™ Red DND-99 (ThermoFisher) for 1 h. After appropriate dye labelling, a fresh medium was added prior to the live cell imaging. Time-lapse recordings of labelled mitochondria and lysosomes were acquired at every 10 s for 100 frames using Zeiss LSM 700 confocal microscope at 63 × oil immersive objective. During imaging, neurons were maintained at 37 °C and 5% CO^2^ in a sealed incubation chamber under the microscope. For a better resolution, less dense axon areas were selected for time-lapse recording. The exposure time was kept short (200 ms for mitochondria and 300 ms for lysosomes) during the imaging to avoid photobleaching and toxicity. Time-lapse image stack was made using Zen software (Zeiss).

### Image Analysis

Time-lapse image data of mitochondria and lysosomes were collected from at least 5–8 DRG explants from 3 different primary cultures. DRG axons which did not displace or move from the base surface during the recording were considered for the analysis. Axonal transport of mitochondria or lysosomes was analyzed using Kymograph plugin in ImageJ software. Briefly, a 100 µm long straight line on an individual axon in proximal to distal direction indicating the cell body position and growth cone area was drawn in the time-lapse image stack. Further, a kymograph was generated for selected area using Kymograph plugin. The kymograph plot represents the mitochondria or lysosomes motility. The vertical line on kymograph indicates the stationary mitochondria or lysosomes while the diagonal line in either direction represents the movement. Since we have drowned a straight line in a fashion where the position of the cell body and axon end is already known, it was easier to determine the direction of anterograde and retrograde movement. Further, the distance travelled by individual mitochondria and lysosomes was measured using plot profile function in ImageJ. Mitochondria or lysosomes travelled more than 10 µm were considered for the analysis. The total, anterograde, and retrograde travelled distance was analyzed. The total number of movements in a selected area was used to analyze the total movement. Spinal cord image analysis was performed using ImageJ software. Briefly, NeuN-positive spinal neurons in the ventral horn bigger than 250μm^2^ were marked using the hand tool in ImageJ to cover the entire cell body area. Further, the integrated density of the protein of interest was taken by switching to the next channel.

### Drug Treatment in Mice and Behavior

Cohorts of *Gan*^*−/−*^;TgPer mice and Gan^±^ mice were produced by crossing TgPer mice [24] with *Gan*^*−/−*^ mice [4] and then Gan^±^;TgPer mice with *Gan*^*−/−*^ mice. Tubastatin A (MedChem Express) drug treatment was given to the *Gan*^*−/−*^;TgPer mice at the age of 12 months. Intraperitoneal injection of TubA (25 mg/kg) as described before for a mouse model of CMT [22] or equal volume of saline (Baxter cooperation) was administered every day for a period of two months. The sex ratio of mice was kept 1:1 for all genotypes. Grid, rotarod, and footprint tests were performed to assess the motor function during the treatment period of *Gan*^*−/−*^;TgPer and Gan^±^ every week (Schematic representation Fig. 6a. Hind limb strength was assessed by grid hang test. Mice were placed on a grill which was gently inverted, the cut off time for grid test was 90 s. For motor performance, mice were trained to run on an accelerating rotarod machine at 3 rpm speed with 0.1 rpm/acceleration. Cut off time for rotarod was set at 300 s and longest latency to fall from rotarod was used for analysis. The footprint test was performed with the use of a homemade test corridor of 90 × 8 cm ramp. Fore and hind limb paws of mice were painted with green and red colors and mice were allowed to walk on a paper on the ramp freely. The stride lengths of fore and hind limbs were measured for analysis. All experiments were approved by the Laval University Animal Care Ethics Committee (Protocol@2020–568) and were in accordance with the guide to care and use of Experimental Animals of Canadian Council on Animal Care.

### Statistics

Statistical analysis was performed using GraphPad Prism 9.4 (GraphPad Software, USA). Comparison between multiple groups were done using One way or two-way ANOVA with Bonferroni’s post-correction. Comparison between two groups was done using unpaired two tailed t-test. P value less than 0.05 was considered significant.

## Results

### Increased IF levels in Cultured DRG Neurons from *Gan*^*−/−*^ Mice

Embryonic DRG explants from *Wt* and *Gan*^*−/−*^ embryos (E15) were cultured and characterized at day 7 in vitro (Fig. 1a). We seeded around 4 to 5 DRG explants on a PDL-coated glass surface without trypsin dissociation. The advantage of DRG explant culture is to allow an organotypic culture that allows ex vivo transfer of entire organ with the complexity of a neuronal network, including the extracellular environment that plays an important role in neural function [25]. Explant DRG neurons grow radially healthier and elongate axons due to the adhesion and organotypic factors. The characterization of DRG neurons were done using Nefl and Prph antibody. Immunofluorescence microscopy revealed higher staining signals for IF proteins (Prph and Nefl) in *Gan*^*−/−*^ neurons than in *Wt* neurons (Fig. 1b). There was also the formation of numerous IF inclusion bodies in *Gan*^*−/−*^ DRG axons, a phenomenon reminiscent of the NF swellings described in human GAN [1, 26]. Western blots of neuronal extracts confirmed the increased levels of Prph and Nefl in *Gan*^*−/−*^ DRG neurons when compared to *Wt* DRG neurons (Fig. 1c).

### Axonal Transport Defects in Cultured *Gan*^*−/−*^ Neurons

**Fig. 2:**
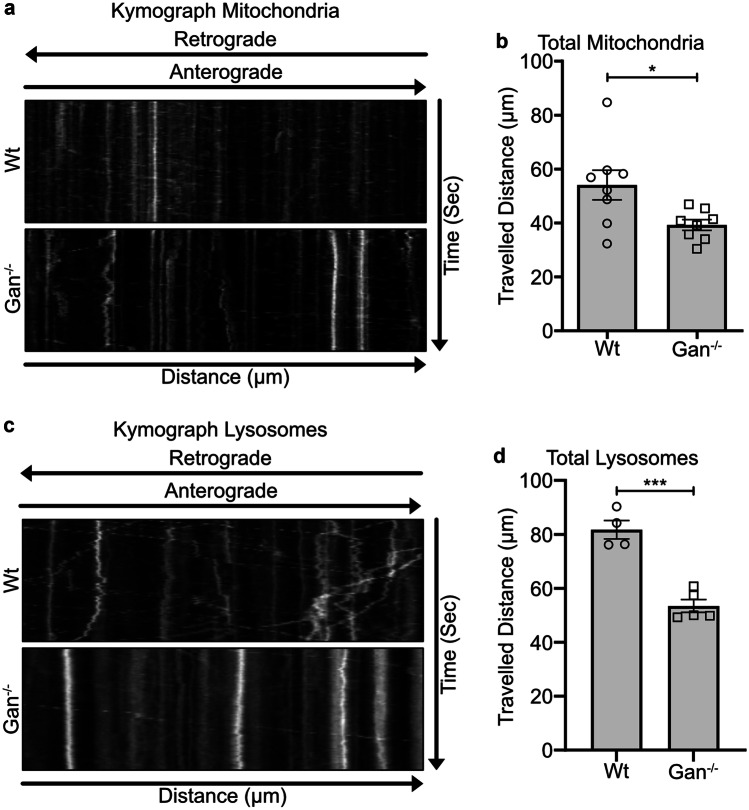
Axonal transport impairment in cultured *Gan*^*−/−*^ DRG neurons. Kymographs (**a**) show mitochondrial motility in *Wt* and *Gan*^*−/−*^ DRG neurons corresponding to the supplementary Fig. 1 video file 1 and 2. Graph (**b**) shows the quantification of mitochondrial movement. The total travelled distance of mitochondria was significantly reduced in *Gan*^*−/−*^ DRG neurons (*Wt* 54.0 ± 5.5 µm, *Gan*^−/−^ 39.2 ± 1.9 µm; p = * 0.0240), n = 8 DRGs explant from 3 independent embryonic cultures. Kymographs (**c**) represent lysosomal motility in *Wt* and *Gan*^*−/−*^ DRG neurons corresponding to the supplementary Fig. 2 video files 3 and 4. Graph (**d**) shows the quantification of total travelled distance of lysosomes in *Wt* and *Gan*^*−/−*^ DRG neurons. Lysosomal travelled distance was significantly reduced in *Gan*^*−/−*^ DRG neurons (*Wt* 81.7 ± 3.4 µm, *Gan*^−/−^ 53.4 ± 2.3 µm; p = *** 0.0001), n = 4 to 5 DRG explants from 3 independent embryonic cultures. Scale bar 100 µm. Kymograph X and Y axis represent distance and time, respectively. Diagonal and vertical line in kymograph represents moving and stationary particles. Data represents ± SEM. Unpaired t-test

Previous studies demonstrated that alterations in IF protein levels and NF organization can provoke defects in fast axonal transport of cellular organelles [8]. This led us to examine by time-lapse microscopy the movement of mitochondria and lysosomes in cultured *Gan*^*−/−*^ DRG neurons with the use of MitoTracker™ and LysoTracker™ (Fisher). MitoTracker™ Green dye stains live mitochondria whereas LysoTracker™ RED DND-099 is an acidotropic fluorescent probe that localizes inside lysosomes. Kymographs generated with straight axons revealed that mitochondria moved less frequently in *Gan*^*−/−*^ DRG neurons than in *Wt* DRG neurons (Fig. 2a). Moreover, the total travelled distance of mitochondria was significantly reduced in cultured *Gan*^*−/−*^ DRG neurons in comparison to *Wt* neurons (Fig. 2b, Supplementary Fig. 1 video file 1 and 2) (*Wt* 54.0 ± 5.5 µm, *Gan*^−/−^ 39.2 ± 1.9 µm; p = * 0.0240). The mitochondrial movements in *Gan*^*−/−*^ DRG neurons were characterized by frequent pauses as compared to *Wt* DRG neurons (Supplementary Fig. 1 video file 1 and 2). The *Gan*^*−/−*^ DRG neurons exhibited a higher number of stationary mitochondria than *Wt* DRG neurons (Supplementary Fig. 1 video file 1 and 2). Changes in mitochondrial morphology are sometimes associated with neuronal damage [27]. Mitochondria detected in cultured *Gan*^*−/−*^ and *Wt* DRG neurons varied in shape (spindle, round, and elongated). However, the mitochondrial morphology did not differ between *Wt* and *Gan*^*−/−*^ DRG neurons.

Like mitochondria, the total travel distance of lysosomes was also significantly reduced in *Gan*^*−/−*^ DRG neurons in comparison to *Wt* DRG neurons (Fig. 2d, Supplementary Fig. 2 video file 3 and 4) (*Wt* 81.7 ± 3.4 µm, *Gan*^−/−^ 53.4 ± 2.3 µm; p = *** 0.0001). Lysosomal kymographs revealed that the number of moving lysosomes was reduced in *Gan*^*−/−*^ DRG neurons when compared to *Wt* DRG neurons (Fig. 2c). The lysosomes were generally stationary in *Gan*^*−/−*^ DRG neurons (Fig. 2c, Supplementary Fig. 2 video file 3 and 4).

### TubA Treatment Enhanced Levels of Acetylated Tubulin and Mitochondria Transport in Cultured *Gan*^*−/−*^ DRG Neurons

**Fig. 3:**
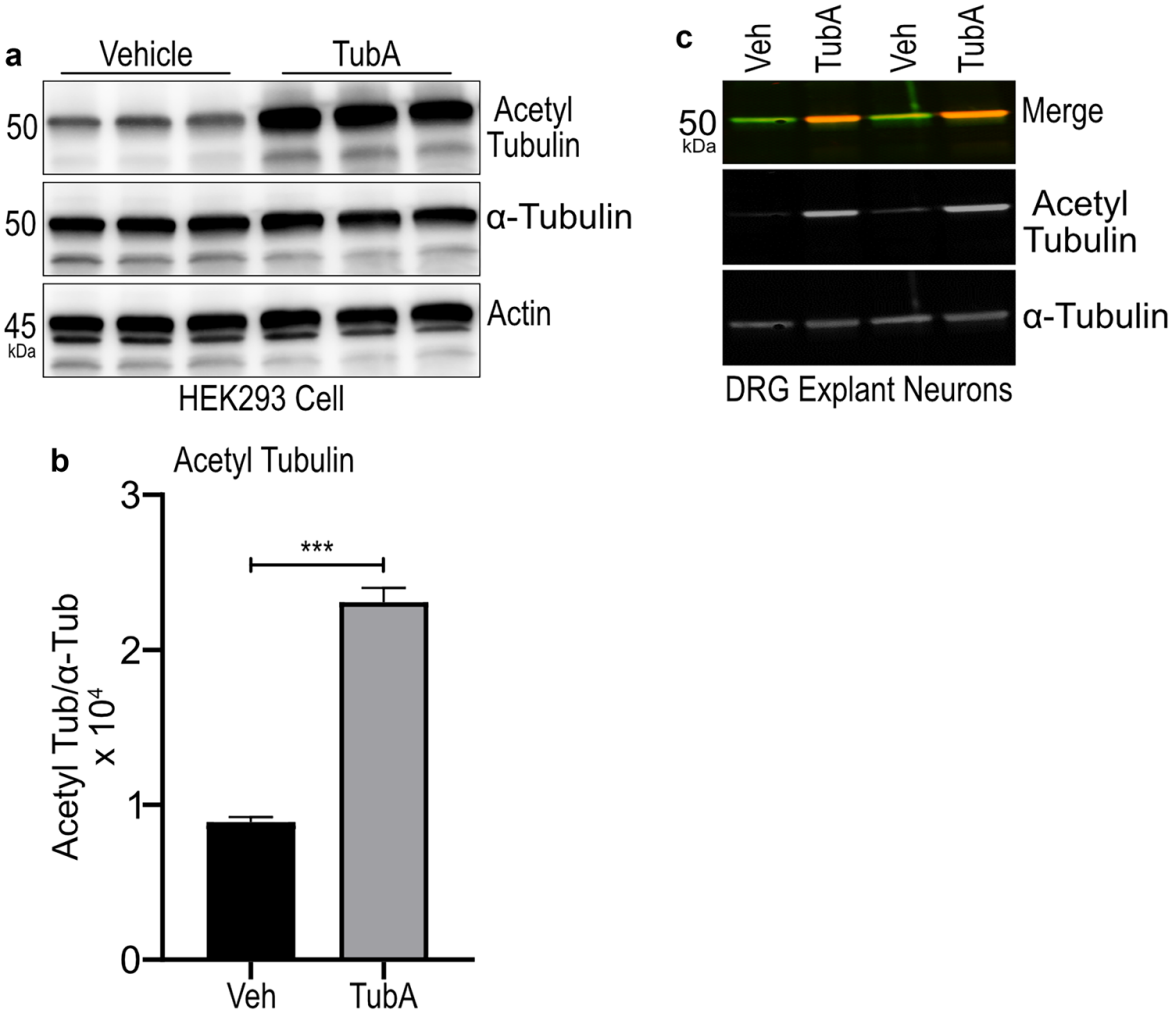
TubA treatment increases acetylated tubulin in HEK293 cells and DRG neurons. Western blots show a significant increase in levels of acetylated tubulin in HEK293 cells. Images (**a**) and (**c**) show western blots using antibodies against acetylated tubulin, α-tubulin, and actin with extracts from HEK293 and DRG neurons, respectively. Graph (**b**) shows the quantification of acetylated tubulin in HEK293 cells normalized with α-tubulin. Treatment with 1 µM TubA increased the levels of acetylated tubulin in HEK293 cells in comparison to vehicle—(DMSO) treated cells (p = *** 0.0001). Data represents ± SEM. Unpaired t-test. n = 3 culture each group

Sustained tubulin acetylation is critical for the maintenance of stable microtubule organization and axonal transport. HDAC6 can deacetylate α-tubulin which results in disorganization of microtubules and impairment of axonal transport [14]. A selective inhibition of HDAC6 enzyme can increase levels of acetylated tubulin, thereby contributing to maintain microtubule organization. TubA, an inhibitor of HDAC6, was reported to enhance acetylated tubulin levels and axonal transport in a mouse model of CMT [22]. Here, we treated HEK293 cells with 1 µM TubA for overnight. Western blots of total protein extracts revealed similar levels of α-tubulin in both TubA-treated and vehicle-treated groups (Fig. 3a). However, the total acetylated tubulin levels were significantly increased in TubA-treated HEK293 cells in comparison to vehicle-treated (DMSO) group (Fig. 3a, b) (p = *** 0.0001). Treatment of cultured *Gan*^*−/−*^ DRG neurons with TubA also resulted in increased levels of acetylated tubulin (Fig. 3c).

**Fig. 4:**
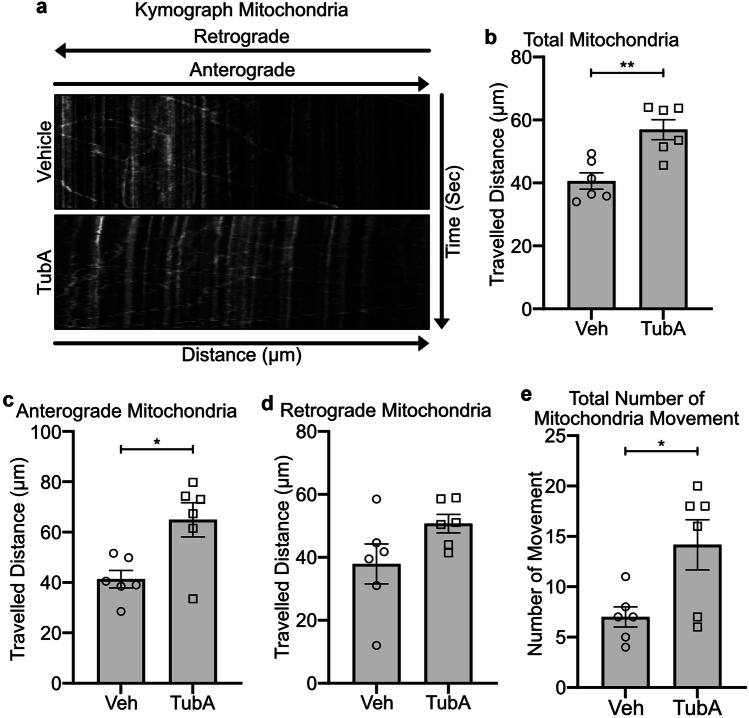
TubA treatment restores mitochondrial transport in cultured *Gan*^*−/−*^ DRG neurons. Total travel distance of mitochondria was restored after 1 µM overnight treatment with TubA. Kymographs (**a**) show mitochondrial motility corresponding to the supplementary Fig. 3 video file 5 and 6 for vehicle and TubA-treated *Gan*^*−/−*^ DRG neurons, respectively. Graph (**b**, **c**, and **d**) show quantification for total, anterograde, and retrograde travelled distance of mitochondria, respectively. The total (Vehicle 40.6 ± 2.5 µm, TubA 56.9 ± 3.1 µm; p = ** 0.0027) and anterograde (Vehicle 41.3 ± 3.4 µm, TubA 64.9 ± 6.7 µm; p = * 0.0112) travelled distances of mitochondria motility were significantly increased (**b** and **c**). Retrograde travelled distance was not significantly changed by TubA treatment (Vehicle 37.9 ± 6.3 µm, TubA 50.7 ± 2.9 µm) changes (**d**), n = 6 DRGs from 3 independent embryonic cultures. Graph (**e**) represents the significant increase in the total movement of mitochondria in TubA-treated *Gan*^*−/−*^ DRG neurons (Vehicle 7.0 ± 1.0, TubA 14.1 ± 2.4; p = * 0.0232), n = 6 DRGs from 3 independent embryonic cultures. Data represents ± SEM. Unpaired t-test. n = 3 culture each group

Overnight treatment of *Gan*^*−/−*^ cultured DRG neurons with 1 µM TubA succeeded in fully restoring the axonal transport of mitochondria (Fig. 4a, b). The total travelled distance of mitochondria in TubA-treated *Gan*^*−/−*^ cultured DRG neurons was significantly increased in comparison to vehicle-treated neurons (Vehicle 40.6 ± 2.5 µm, TubA 56.9 ± 3.1 µm; p = ** 0.0027) (Fig. 4b, Supplementary Fig. 3 video file 5 and 6). The kymographs indicated that mitochondria travelled for long distance in *Gan*^*−/−*^ DRG neurons treated with TubA whereas mitochondria moved for short distance in vehicle-treated neurons (Fig. 4a). We have examined the impact of TubA treatment on the direction of mitochondrial movement. As shown in Fig. 4c, TubA- treatment significantly increased the anterograde travel distance of mitochondria in *Gan*^*−/−*^ DRG neurons (Vehicle 41.3 ± 3.4 µm, TubA 64.9 ± 6.7 µm; p = * 0.0112). There was a tendency of increase in retrograde travel distance of mitochondria, but it was not significant (Vehicle 37.9 ± 6.3 µm, TubA 50.7 ± 2.9 µm) (Fig. 4d). The kymographs and time lapse recording suggest that the overall motility of mitochondria toward the anterograde direction was increased by TubA treatment. However, the time lapse images were acquired at every 10 s for 100 frames, reflecting only the movement not the velocity of organelles. (Supplementary Fig. 3 video file 5 and 6). It is noteworthy that the total number of moving mitochondria within 1,000 s in selected axon length of 100 µm was also significantly increased in TubA-treated group (Vehicle 7.0 ± 1.0, TubA 14.1 ± 2.4; p = * 0.0232) (Fig. 4e). Furthermore, the morphology of mitochondria did not change (Supplementary Fig. 3 video file 5 and 6).

### TubA Treatment Restored Lysosomal Transport in *Gan*^*−/−*^ Cultured DRG Neurons

**Fig. 5:**
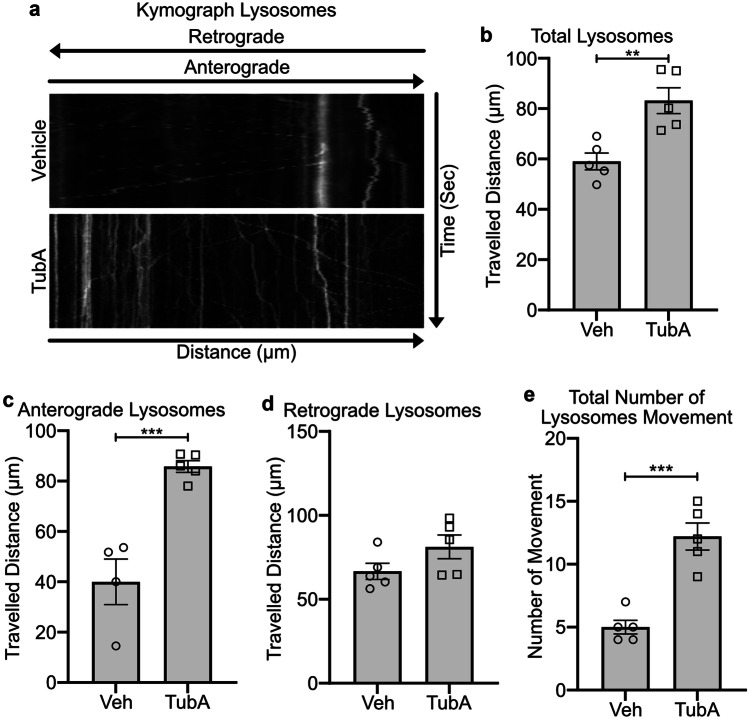
TubA treatment restored lysosome transport in cultured *Gan*^*−/−*^ DRG neurons. Total travelled distance of lysosomes was restored after 1 µM overnight treatment of TubA. Kymographs (**a**) show lysosomal motility corresponding to the supplementary Fig. 4 video file 7 and 8 for vehicle and TubA-treated *Gan*^*−/−*^ DRG neurons, respectively. Graphs (**b**, **c**, and **d**) show quantification for total, anterograde, and retrograde travelled distance of lysosomes, respectively. The total (Vehicle 59.0 ± 3.3 µm TubA 83.1 ± 5.1 µm; p = ** 0.0044) and anterograde (Vehicle 39.9 ± 9.0 µm, TubA 85.8 ± 2.3 µm; p = *** 0.0009) travelled distances of lysosomes were significantly increased (**b** and **c**). Retrograde travelled distances (**d**) do not show any significant changes (Vehicle 66.6 ± 4.8 µm, TubA 81.2 ± 7.0 µm), n = 4 ~ 5 DRGs from 3 independent embryonic cultures. Graph (**e**) represents the significant increase in the total movement of lysosomes in TubA-treated *Gan*^*−/−*^ DRG neurons (Vehicle 5.0 ± 0.5, TubA 12.2 ± 1.0; p = *** 0.0003), n = 4 ~ 5 DRGs from 3 independent embryonic cultures. Data represents ± SEM. Unpaired t-test. n = 3 culture each group

Overnight treatment of cultured *Gan*^*−/−*^ DRG neurons with 1 µM TubA restored the total distance travelled by lysosomes (Vehicle 59.0 ± 3.3 µm, TubA 83.1 ± 5.1 µm; p = ** 0.0044) (Fig. 5a, b, Supplementary Fig. 4 video file 7 and 8). The kymographs of lysosomes revealed movement patterns like mitochondria with long persistent movement and less abrupt pauses in TubA-treated *Gan*^*−/−*^ DRG neurons (Fig. 5a). Further, the travelled distance of lysosomes was significantly increased in the anterograde direction (Vehicle 39.9 ± 9.0 µm, TubA 85.8 ± 2.3 µm; p = *** 0.0009) (Fig. 5c) but not in the retrograde direction in *Gan*^*−/−*^ DRG neurons (Vehicle 66.6 ± 4.8 µm, TubA 81.2 ± 7.0 µm) (Fig. 5d). Moreover, the total number of lysosome movements within 1,000 s in selected axon length of 100 µm was also significantly increased in TubA-treated *Gan*^*−/−*^ cultured DRG neurons in comparison with vehicle-treated group (Vehicle 5.0 ± 0.5, TubA 12.2 ± 1.0; p = *** 0.0003) (Fig. 5e).

### TubA Treatment Ameliorated the Gait of *Gan*^*−/−*^;TgPer Mice

The observation that TubA treatment rescued axonal transport defects in cultured *Gan*^*−/−*^ DRG neurons led us to test this drug in a new mouse model of GAN exhibiting overt phenotypes, the *Gan*^*−/−*^;TgPer mouse. The *Gan*^*−/−*^;TgPer mice were generated by crossing transgenic mice overexpressing peripherin (Prph) with mice knockout for *Gan.* The gigaxonin deficiency in the TgPer mice caused large NF accumulations in neuronal subsets in the brain and spinal cord, and it provoked neuronal dysfunction and degeneration during aging [23].

**Fig. 6:**
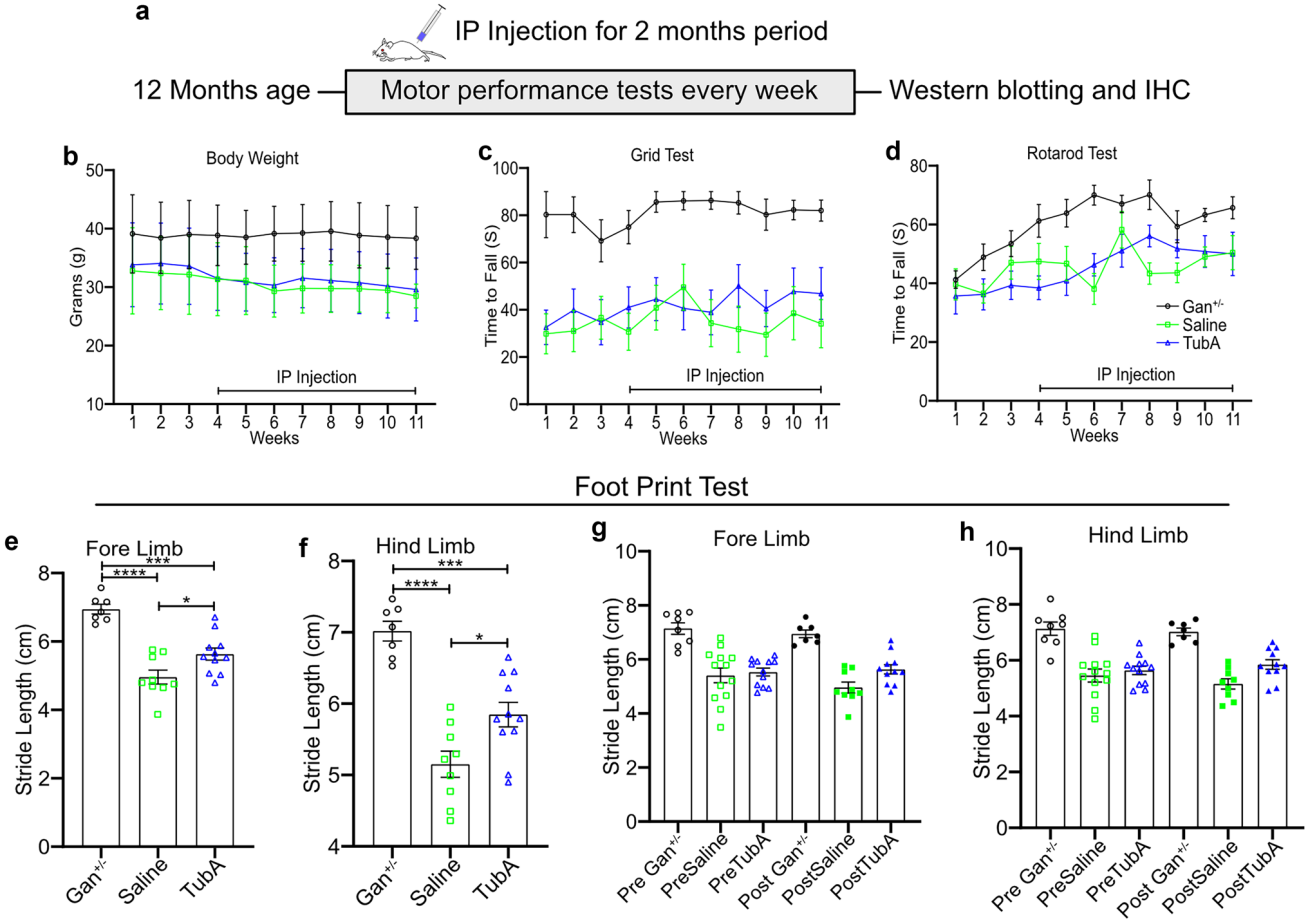
Effects of TubA treatment on motor dysfunction of 12-month-old *Gan*^*−/−*^;TgPer mice. Top panel shows schematic representation of the drug treatment period (**a**). Graph panels (**b**, **c**, and **d**) represent body weight, grid, and rotarod test in control (Gan^±^), saline- and TubA-treated *Gan*^*−/−*^;TgPer mice. There was a tendency for the TubA-treated *Gan*^*−/−*^;TgPer mice to perform better than the saline-treated *Gan*^*−/−*^;TgPer mice in the grid and rotarod tests between 4 and 5 weeks of treatment, albeit the changes were not significant. Graph panels (**e**) and (**f**) show the quantification of fore- and hind-limb stride lengths of footprint test at the end of treatment period. TubA-treated mice (5.6 cm and 5.8 cm respectively) exhibited a significant increase in the stride length of fore- and hind-limbs in comparison to saline-treated mice (4.9 cm and 5.1 cm respectively). p = * 0.0344 (Saline vs TubA) in fore limb; * 0.0186 (Saline vs TubA) in hind limb. Control age-matched Gan^±^ mice had a significantly higher stride length of fore- and hind-limbs (6.9 cm and 7.0 cm respectively) than saline- and TubA-treated mice. p = **** 0.0001, *** 0.0001 (Control vs Saline, Control vs TubA) in forelimbs; **** 0.0001, *** 0.0003 (Control vs Saline, Control vs TubA) in hindlimbs. Graph panels (**g** and **h**) represent the pre and post-treatment comparison of fore- and hind-limb stride lengths between control (Gan^±^), saline, and TubA mice. n = 8 ~ 12 mice per group. Data represents ± SEM, one- and two-way ANOVA analysis of variance with post-Bonferroni’s multiple comparison test

TubA was administered daily intraperitoneally at 25 mg/kg to *Gan*^*−/−*^;TgPer mice, starting at the age of 12 months. Rotarod, grid, and footprint tests were performed to assess the motor performance and gait impairment during the treatment period (Fig. 6a). Over a period of 11 weeks, the body weight of treated- and saline-groups remained stable (Fig. 6b). The results of the grid test revealed a tendency for the TubA-treated *Gan*^*−/−*^;TgPer mice to perform slightly better than the vehicle-treated *Gan*^*−/−*^;TgPer mice after 4 weeks of treatment (Fig. 6c). However, the difference in the time to fall was not statistically significant. With the rotarod test, there was also a slight increase in latency to fall in the TubA-treated *Gan*^*−/−*^;TgPer mice in comparison to saline-treated mice between 4 and 5 weeks of treatment (Fig. 6d). However, at the end of the treatment period, the groups did not perform differently. As expected, the age-matched heterozygous Gan^±^ mice, which are normal, were performing much better in the grid and rotarod tests than the *Gan*^*−/−*^;TgPer mice (Fig. 6c, d).

The 12-month-old *Gan*^*−/−*^;TgPer mice exhibited gait abnormalities using the footprint test with shorter stride lengths of fore- and hind-limbs than age-matched heterozygous Gan^±^ mice (Fig. 6e–h). It is noteworthy that TubA treatment of *Gan*^*−/−*^;TgPer mice (TubA fore- and hind-limb 5.6 cm and 5.8 cm, respectively) increased significantly the stride lengths of limbs when measured at the end of treatment when compared to saline-treated *Gan*^*−/−*^;TgPer mice (Saline fore- and hind-limb 4.9 cm and 5.1 cm, respectively), p value 0.0344 and 0.0186, respectively (Fig. 6e, f). Yet, the fore- and hind-limb stride lengths of TubA-treated *Gan*^*−/−*^;TgPer mice were shorter than those of control Gan^±^ mice (6.9 cm and 7.0 cm respectively), p value 0.0001. The pre-treatment and post-treatment data analyses of footprint test (Fig. 6g, h) suggest that TubA preserved the gait performance of *Gan*^*−/−*^;TgPer mice during the treatment period. In contrast, the saline-treated *Gan*^*−/−*^;TgPer mice exhibited a decline in stride lengths of fore- and hind-limbs.

### TubA Treatment Increased the Levels of Acetylated Tubulin in the Sciatic Nerve of *Gan*^*−/−*^;TgPer Mice

**Fig. 7:**
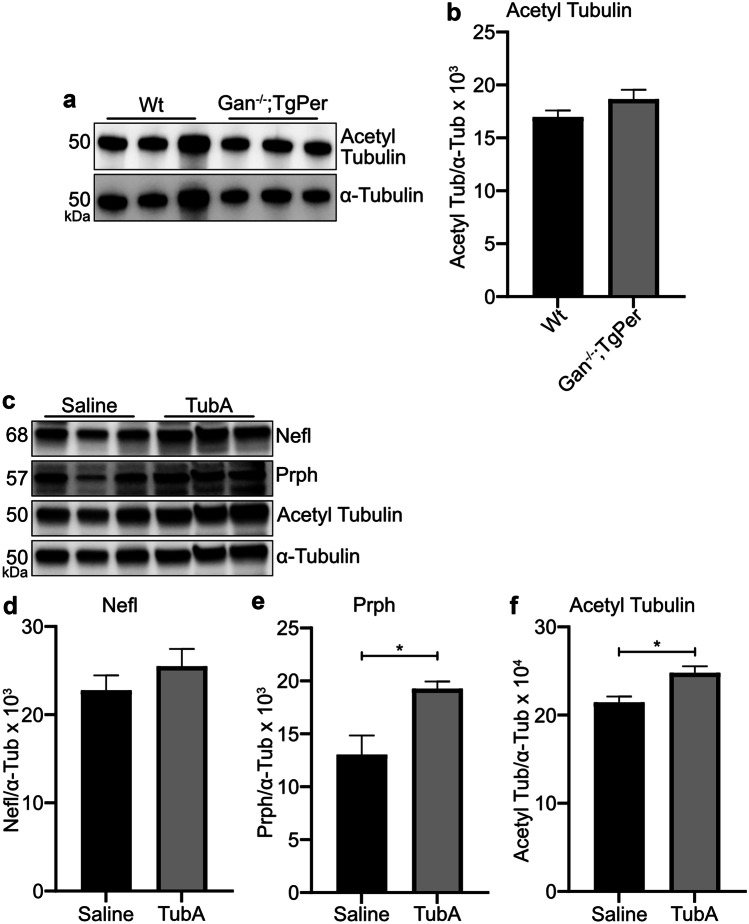
TubA treatment increased levels of acetylated tubulin and Prph in the sciatic nerve of *Gan*^*−/−*^;TgPer mice. Image panel (**a**) shows the western blot of acetyl tubulin and α-tubulin from the sciatic nerve of Wt and *Gan*^*−/−*^;TgPer mice. Quantification of acetylated tubulin levels did not show any significant change between Wt and *Gan*^*−/−*^;TgPer mice, Graph (**b**). p = ns 0.1503 (Wt vs *Gan*^*−/−*^;TgPer). Image panel (**c**) represents the western blots for Nefl, Prph, acetyl tubulin, and α-tubulin, respectively from the sciatic nerve of saline- and TubA-treated mice. Graphs (**d**, **e**, and **f**) represent the quantification of Nefl, Prph, and acetyl tubulin, respectively. The TubA-treated mice exhibited a significant increase in levels of Prph protein (~1.4 folds) in comparison to saline-treated mice, graph (**e**). n = * 0.0325 (Saline vs TubA). The acetylated tubulin levels in the sciatic nerve of TubA-treated mice were significantly increased in comparison with saline-treated mice, graph (**f**). p = * 0.0304 (Saline vs TubA). Quantification of Nefl levels did not reveal significant differences between the groups, graph (**d**). p = ns 0.3533 (Saline vs TubA). Data represents ± SEM. Unpaired t-test. n = 3 culture each group

The axonal transport deficits observed in the *Gan*^*−/−*^ cultured DRG neurons (Fig. 2) incited us to examine the levels of acetylated tubulin in the *Gan*^*−/−*^;TgPer mice. Western blotting of total protein extracts from the sciatic nerve was carried out. As shown in Fig. 7a, b, the levels of acetylated tubulin in the sciatic nerve of *Gan*^*−/−*^;TgPer mice were similar to those found in Wt mice (Fig. 7a, b). So, a gigaxonin deficiency did not alter the acetylation of tubulin in this mouse model of GAN. However, when treated with TubA, the *Gan*^*−/−*^;TgPer mice exhibited a significant increase in levels of acetylated tubulin as revealed by the western blot analysis of protein extracts from the sciatic nerve ( (Fig. 7c, f). Remarkably, the TubA treatment of *Gan*^*−/−*^;TgPer mice led to a significant increase in levels of Prph (~1.4 folds) in the sciatic nerve when compared to saline-treated *Gan*^*−/−*^;TgPer mice. (Fig. 7c–e). The TubA treatment did not affect significantly the levels of Nefl in the sciatic nerve (Fig. 7d).

### TubA Treatment Reduced the Abnormal Accumulation of IF Proteins in Spinal Neurons

**Fig. 8:**
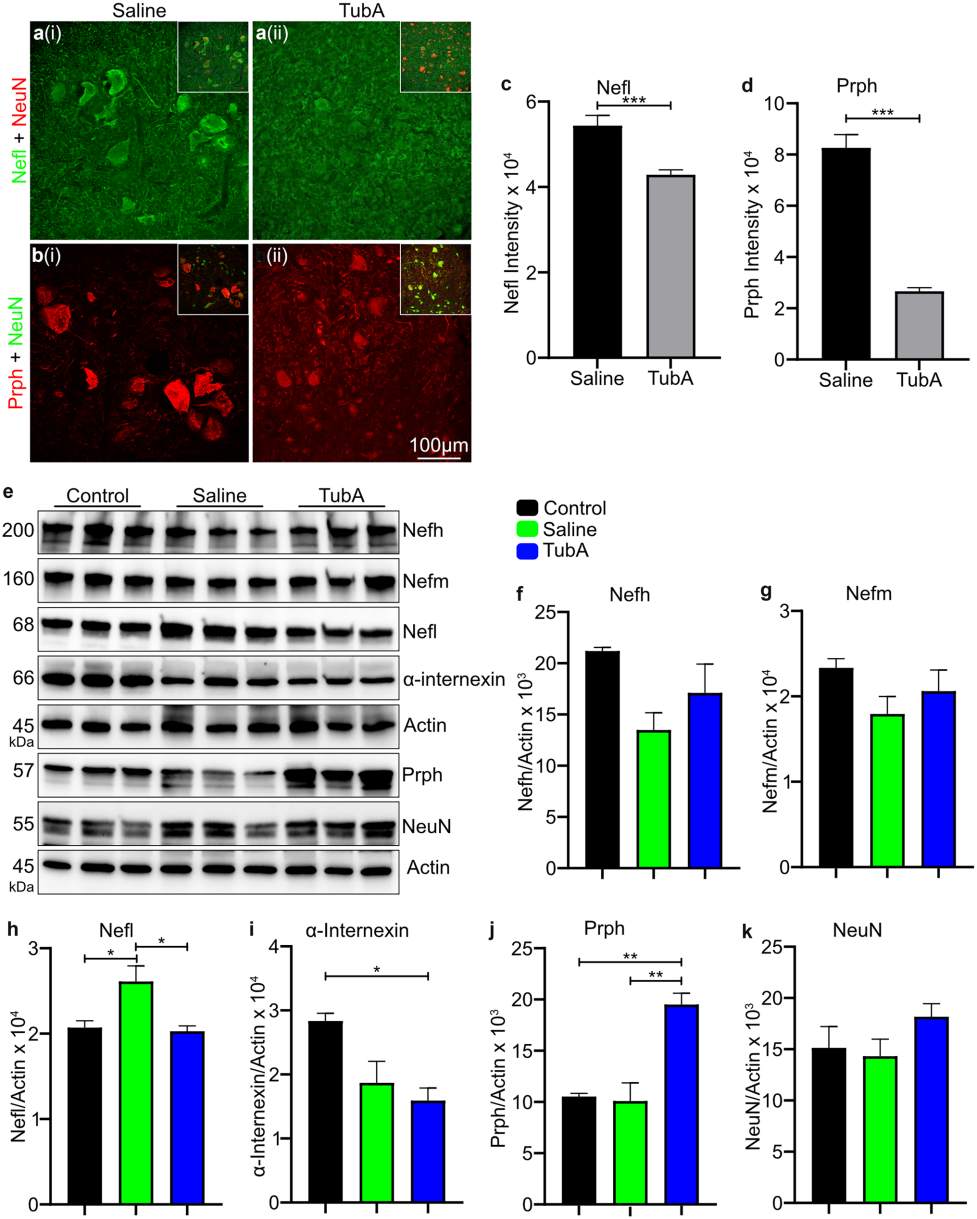
TubA treatment of *Gan*^*−/−*^;TgPer mice reduced the perikaryal IF accumulations in the spinal cord with a concomitant increase of Prph in neuronal projections. Image panel (a and b) represent the immunostaining of Nefl and Prph, respectively. Colors green, red, and blue represent Nefl, NeuN, and DAPI respectively for the image panel (**ai**-**ii**). Color green, red, and blue represent NeuN, Prph, and DAPI respectively for the image panel (**bi**-**ii**). Original magnification 25x. Scale bar 100 μm. Inlet in the image panel a and b represents the merge image. Graphs c and d show the quantification for the Nefl and Prph signal intensity in the cell body area of spinal neurons (larger than 250μm^2^) of saline and TubA-treated mice, respectively. The signal intensities for Nefl and Prph proteins in neuronal cell bodies of TubA-treated mice were significantly decreased in comparison to saline-treated mice *Gan*^*−/−*^;TgPer mice, graph (**c** and **d**), respectively. p = *** 0.0001 for Nefl and Prph (Saline- vs TubA-treated mice). The signal intensities of spinal neurons larger than 250µm^2^ in the ventral horn of the lumbar region were taken from at least 6 spinal cord sections, n = 3 mice per group. Data represent ± SEM. Unpaired t-test. Image panel (**e**) shows the western blot of total protein extract for Nefh, Nefm, Nefl, α-internexin, Prph, NeuN, and actin in the spinal cord of control, saline, and TubA-treated mice. Graphs (**f** to **k**) represent the quantification of Nefh, Nefm, Nefl, α-internexin, Prph, and NeuN proteins in the spinal cord of control (Gan^±^) mice, saline-treated- and TubA-treated *Gan*^*−/−*^;TgPer mice, respectively. The Nefl protein levels of saline-treated *Gan*^*−/−*^;TgPer mice were significantly higher than the control (Gan^±^) mice and TubA-treated *Gan*^*−/−*^;TgPer mice, graph (**h**). p = *0.0433 (Control vs Saline), 0.0319 (Saline vs TubA). α-internexin levels in spinal cord of control (Gan^±^) mice were also significantly higher (~2 fold) than the TubA-treated *Gan*^*−/−*^;TgPer mice, graph (**i**). p = * 0.0296 (Control vs TubA). In contrast, the Prph levels (~2 fold) of TubA-treated mice were significantly higher than the control (Gan^±^) mice and saline-treated *Gan*^*−/−*^;TgPer mice, graph (**j**). p = ** 0.0059 (Control vs TubA-treated *Gan*^*−/−*^;TgPer mice), 0.0047 (Saline-treated vs TubA-treated *Gan*^*−/−*^;TgPer mice). The levels of Nefh, Nefm, and NeuN did not vary significantly between the different groups, graphs (**f**, **g**, and **k**). Data represent ± SEM, one-way ANOVA analysis of variance with post-Bonferroni’s multiple comparison test

Immunofluorescence microscopy of the spinal cord revealed that there was a significant decrease in the signal intensity for Nefl and Prph proteins in the perikaryon of spinal neurons from *Gan*^*−/−*^;TgPer mice treated with TubA when compared to saline-treated *Gan*^*−/−*^;TgPer mice (Fig. 8a–d). Thus, the TubA treatment reduced the abnormal perikaryal swellings of spinal neurons in *Gan*^*−/−*^;TgPer mice. It is noteworthy that the TubA treatment resulted in a transfer of Prph signals from cell bodies to neuronal projections (Fig. 8bi, bii). In fact, western blot data revealed that the Prph levels in the spinal cord extracts of *Gan*^*−/−*^;TgPer mice were increased by 2 folds as a result of TubA treatment (Fig. 8e, j). The combined results suggest that TubA treatment mitigated the formation of large IF accumulations in neuronal perikaryal by boosting the transport of Prph into projections and peripheral nerve axons. The TubA treatment did not affect the levels of Nefh and Nefm in the spinal cord (Fig. 8e–g). However, the Nefl levels in the spinal cord of TubA-treated *Gan*^*−/−*^;TgPer mice were slightly reduced (Fig. 8e, h), a phenomenon probably due to the competing assembly of excess Prph with Nefh and Nefm to form IF structures.

## Discussion

Gigaxonin plays a key role in maintaining the turnover of IF proteins by recruiting them towards Cul3 ubiquitin to undergo proteasomal-mediated degradation [5]. So, it is well established that a deficiency in gigaxonin, a phenomenon associated with GAN, can cause accumulations of IF proteins [1, 2, 4]. However, the effects of IF disorganization on neuronal function in GAN remain unknown. Here, our results support the view that NF accumulations in GAN might contribute to axonal transport impairment of organelles such as mitochondria and lysosomes. To address this topic, we used cultured embryonic DRG neurons derived from *Gan*^*−/−*^ mice [4]. Immunoblotting of cell extracts confirmed the excess levels of Nefl and Prph proteins in *Gan*^*−/−*^ DRG neurons lacking gigaxonin. Accordingly, immunofluorescence microscopy revealed an increase of immunostaining for Nefl and Prph in *Gan*^*−/−*^ neurons as compared to *Wt* neurons (Fig. 1). In addition, microscopy inclusion bodies positive for Nefl and Prph were abundant in *Gan*^*−/−*^ axons (Fig. 1b).

To further address whether the lack of gigaxonin may affect the fast axonal transport of organelles, we generated kymographs by time-lapse microscopy of DRG axons with the use of MitoTracker^FM^ and LysoTracker^FM^ dye stains. The kymograph analyses revealed that a deficiency in gigaxonin caused anterograde axonal transport defects of mitochondria and lysosomes. Thus, the travelled distance and the number of movements of these organelles were substantially reduced in the absence of gigaxonin. These results are in line with the view that NF accumulations in *Gan*^*−/−*^ DRG neurons can impair the motility of mitochondria [9]. It is conceivable that Ifs may serve as docking sites for mitochondria and lysosomes, thereby interfering with their anterograde transport. Nonetheless, it is remarkable that the retrograde transport remained unaffected in *Gan*^*−/−*^ neurons. DRG neurons overexpressing Prph in context of Nefl^*−/−*^ exhibited an increased proportion of retrograde mitochondrial movements [8]. How NF disorganization may affect selective kinesin-mediated transport remains to be elucidated. There is possibility that deposits of excess NFs may sequester kinesin motors shared by various organelles. This idea is supported by the report that kinesin accumulated in axonal swellings induced by intoxication with beta, beta’-iminodipropionitrile (IDPN) [28].

Our results demonstrate a rescue of axonal transport defects in *Gan*^*−/−*^ neurons with TubA, an inhibitor of HDAC6. TubA treatment led to enhanced levels of acetylated tubulin in cultured *Gan*^*−/−*^ DRG neurons (Fig. 3) and it restored the anterograde transport of mitochondria and lysosomes due to gigaxonin deficiency (Figs. 4 and 5). It is well established that acetylation is a post-translational modification of α-tubulin that facilitates kinesin binding onto microtubules [11]. In a mouse model of CMT neuropathy bearing a heat shock HSPB1 gene mutant, pharmacological inhibition of HDAC6 also succeeded in rescuing axonal defects and in ameliorating disease phenotypes [22]. Inhibition of HDAC6 was also found to induce mitochondrial fusion and autophagy in mutant huntingtin striatal neurons [29].

Furthermore, we tested TubA in a new animal model of GAN, the *Gan*^*−/−*^;TgPer mouse, which is lacking gigaxonin in context of overexpression of a Prph transgene. The *Gan*^*−/−*^;TgPer mice develop large IF accumulations made up of NF proteins and of Prph, causing swelling of spinal neurons. They also develop IF inclusion bodies in brain neuronal subsets and giant axons in peripheral nerves [23]. As shown in Fig. 6, the *Gan*^*−/−*^;TgPer mice at 12 months of age exhibited severe motor dysfunction reflected by a poor performance in the grid test, rotarod test, and gait analysis. When initiated at 12 months of age, the intraperitoneal injection of TubA in *Gan*^*−/−*^;TgPer mice led to significant amelioration of the gait abnormality (Fig. 6e, f). The gait abnormality test was based on the measurement of the stride length of footprints after a period of 8 weeks of treatment. It is noteworthy that TubA treatment did not totally restore the gait to normal as measured in age-match Gan^±^ mice. In fact, our analysis of footprints before and after treatment suggests that TubA prevented further gait decline after beginning of treatment (Fig. 6g, h). This is not unexpected as the *Gan*^*−/−*^;TgPer mice exhibit a 25% loss of motor neurons at 12 months of age [23] Despite beneficial effects of TubA (Figs. 4 and 5), it was likely too late at this age for a treatment to promote full recovery of motor function. This may explain the modest effects of the drug on the performance of *Gan*^*−/−*^;TgPer mice in the grip and rotarod tests. There was a tendency for better performance in these tests after 4 and 5 weeks of treatment but it remained not statistically significant (Fig. 6c, d). Alternatively, TubA may not have reached a sufficient level of target engagement in vivo for maximal effects and we cannot exclude that other HDAC6 substrates than tubulin could mediate the effects of TubA in vivo [30, 31].

The levels of acetylated tubulin were significantly increased in the sciatic nerve of TubA-treated *Gan*^*−/−*^;TgPer mice when compared to saline-treated *Gan*^*−/−*^;TgPer mice (Fig. 7f). It should be noted that the lack of gigaxonin did not alter the levels of acetylated tubulin in peripheral nerves of the *Gan*^*−/−*^;TgPer mice when compared to normal Wt mice (Fig. 7a, b). So, evidence suggests that the abnormal IF accumulations in this mouse model of GAN are not due to transport defects mediated by alterations in levels of acetylated tubulin. Yet, treatment with TubA in this mouse model boosted the acetylation of tubulin at levels sufficient to alleviate the abnormal IF accumulations in neurons (Fig. 8a, b). TubA treatment reduced the immunofluorescence of Prph and Nefl in neuronal perikaryal in the spinal cord whereas it increased Prph levels in the spinal cord (Fig. 8j) and sciatic nerve (Fig. 7e). Our interpretation of these results is that TubA treatment enhanced, via tubulin acetylation, the transport of Prph into axons resulting in a redistribution of Prph proteins from perikaryal IF accumulations into projections and axonal compartments of spinal cord and peripheral nerve.

Our results suggest that GAN disease evolves with NF accumulations and axonal transport defects starting early, at embryonic stage. Thus, time-lapse microscopy of cultured embryonic *Gan*^*−/−*^ DRG neurons revealed impairment of anterograde movements of mitochondria and lysosomes, a phenomenon which may progressively alter axon integrity and synapse homeostasis [32]. Treatment with TubA, an inhibitor of HDAC6 aiming to enhance tubulin acetylation, succeeded in rescuing axonal transport of these organelles in cultured neurons. Furthermore, in a mouse model of GAN based on Prph overexpression in absence of gigaxonin, TubA treatment starting at 12-month-old mitigated gait defects, and it reduced the abnormal IF accumulations by boosting acetyl tubulin levels and transport of Prph proteins into axons. In future, it would be of interest to initiate chronic administration of TubA in young mice with a monitoring of symptom phenotypes and of pathological changes during aging. In conclusion, our results suggest that clinical studies should be considered to assess the potency of HDAC6 inhibitors in human GAN and possibly with therapeutic intervention initiated at young age.

## Supplementary Information

Below is the link to the electronic supplementary material.

Supplementary file1 (PPTX 25460 KB)

## Data Availability

The authors declare that the data supporting the findings of this study are available within the paper and its supplementary files. Should any raw data files be needed, they are available from the corresponding author upon reasonable request.
