# Supplementary material for: Axonal Transport Defect in Gigaxonin Deficiency Rescued by Tubastatin A
Source: Neurotherapeutics. 2023 Jun 2;20(4):1215–28. doi: 10.1007/s13311-023-01393-1 (PMC10457258; doi:10.1007/s13311-023-01393-1)
Supplement: Supplementary file 1 — Supplementary file1 (PPTX 25460 KB) [file 13311_2023_1393_MOESM1_ESM.pptx]

## Slide 1
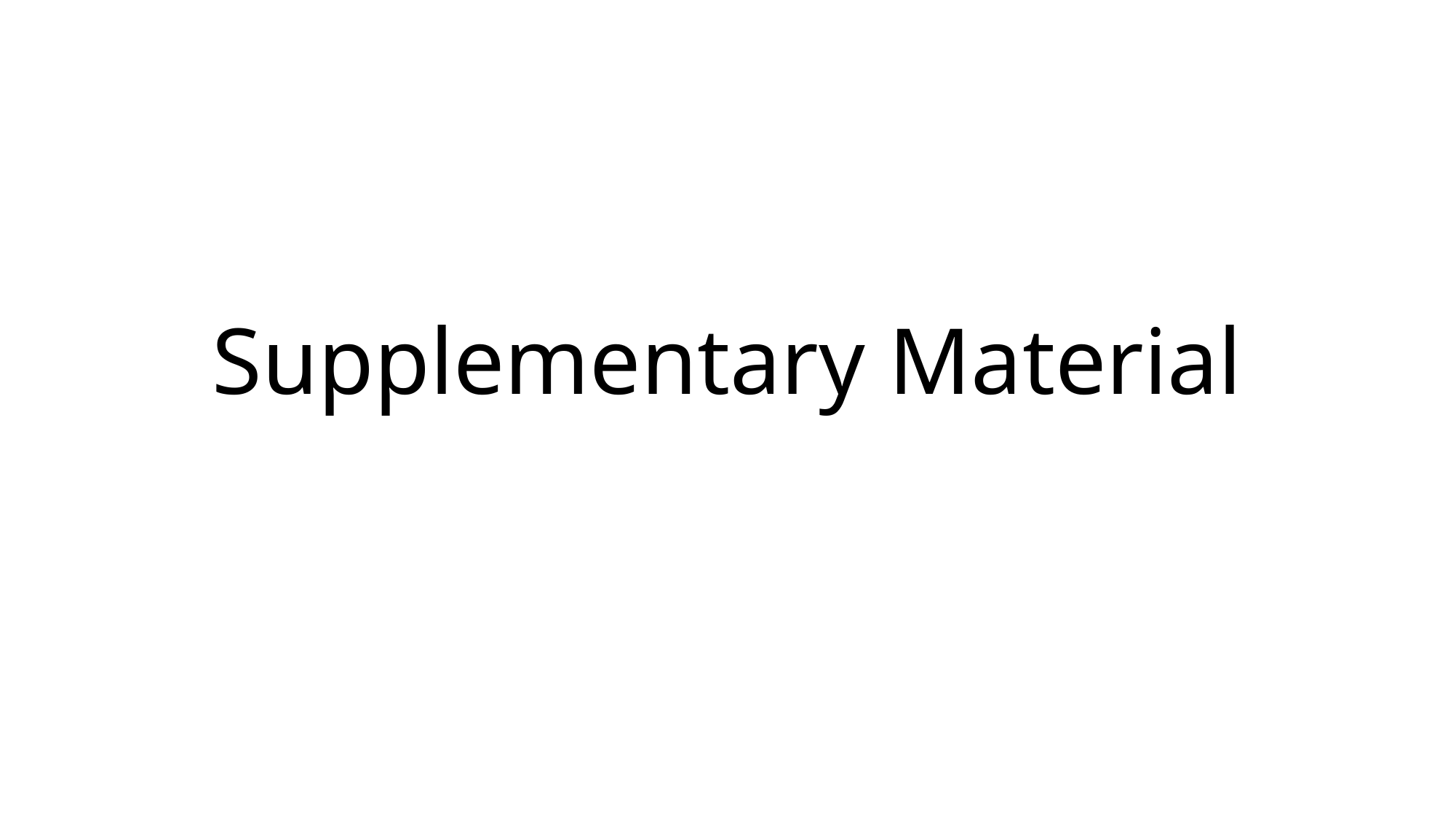

# Supplementary Material

## Slide 2
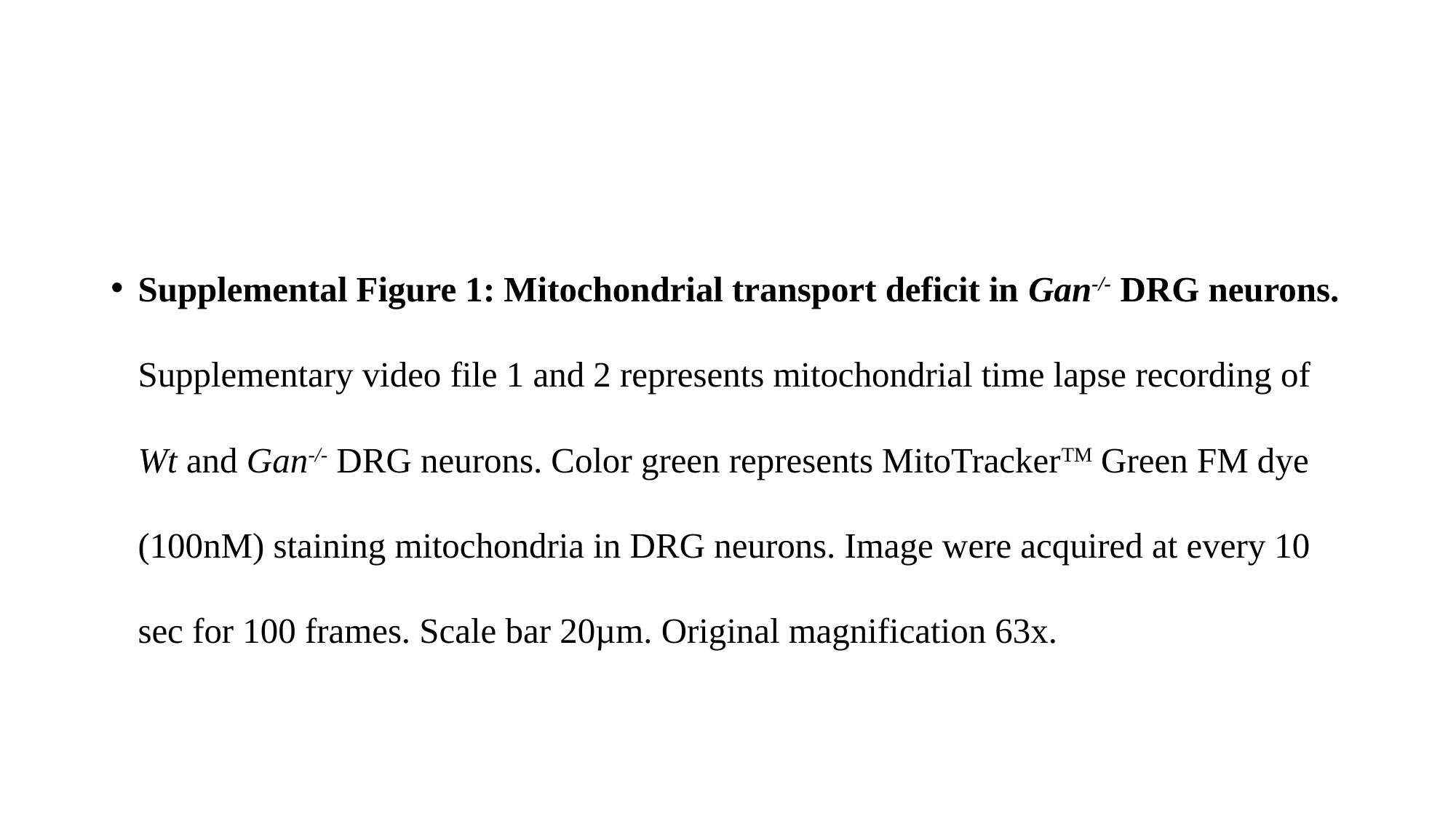

#
Supplemental Figure 1: Mitochondrial transport deficit in Gan-/- DRG neurons. Supplementary video file 1 and 2 represents mitochondrial time lapse recording of Wt and Gan-/- DRG neurons. Color green represents MitoTrackerTM Green FM dye (100nM) staining mitochondria in DRG neurons. Image were acquired at every 10 sec for 100 frames. Scale bar 20µm. Original magnification 63x.

## Slide 3
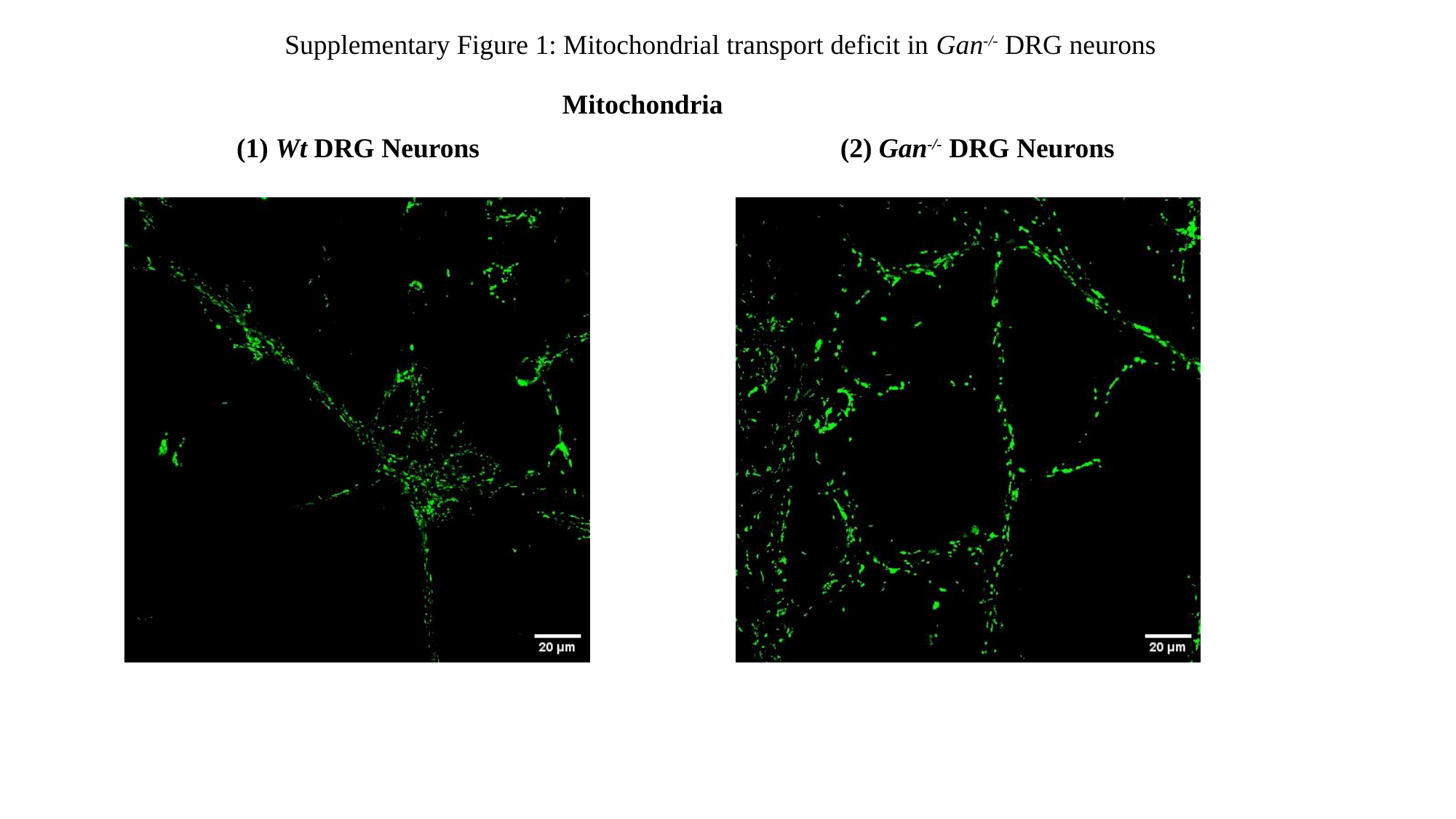

Supplementary Figure 1: Mitochondrial transport deficit in Gan-/- DRG neurons
Mitochondria
(1) Wt DRG Neurons
(2) Gan-/- DRG Neurons

## Slide 4
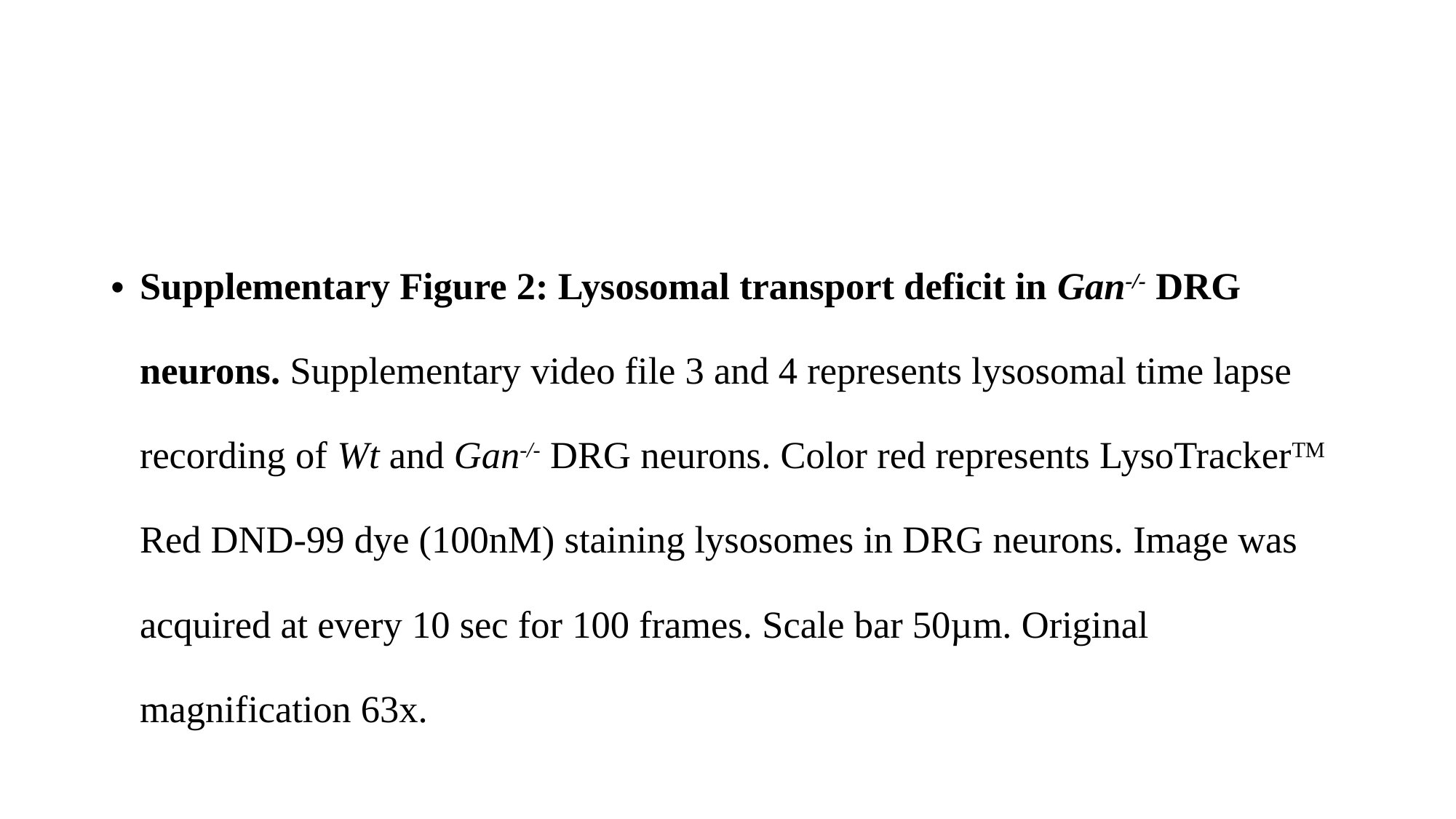

#
Supplementary Figure 2: Lysosomal transport deficit in Gan-/- DRG neurons. Supplementary video file 3 and 4 represents lysosomal time lapse recording of Wt and Gan-/- DRG neurons. Color red represents LysoTrackerTM Red DND-99 dye (100nM) staining lysosomes in DRG neurons. Image was acquired at every 10 sec for 100 frames. Scale bar 50µm. Original magnification 63x.

## Slide 5
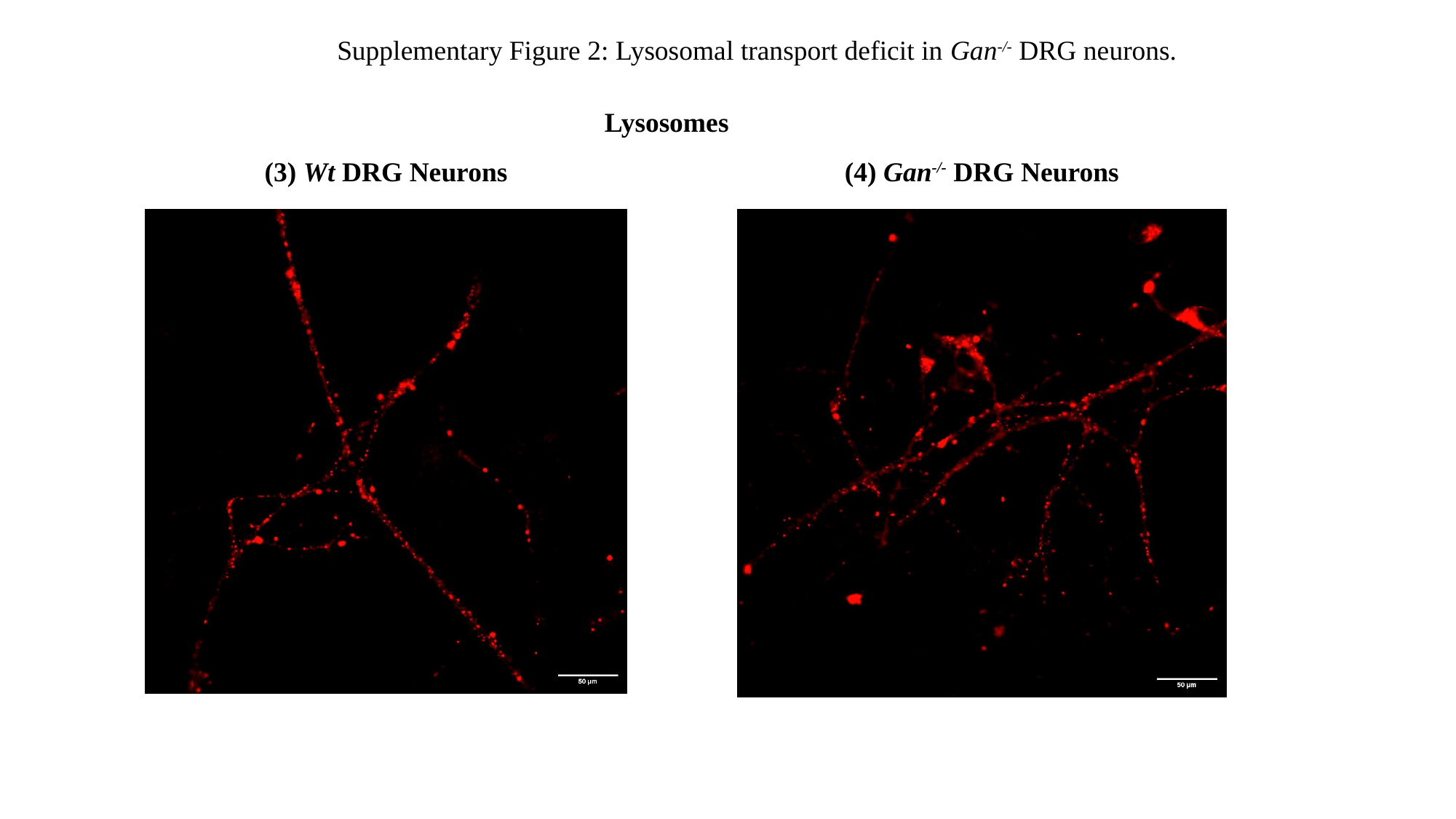

Supplementary Figure 2: Lysosomal transport deficit in Gan-/- DRG neurons.
Lysosomes
(3) Wt DRG Neurons
(4) Gan-/- DRG Neurons

## Slide 6
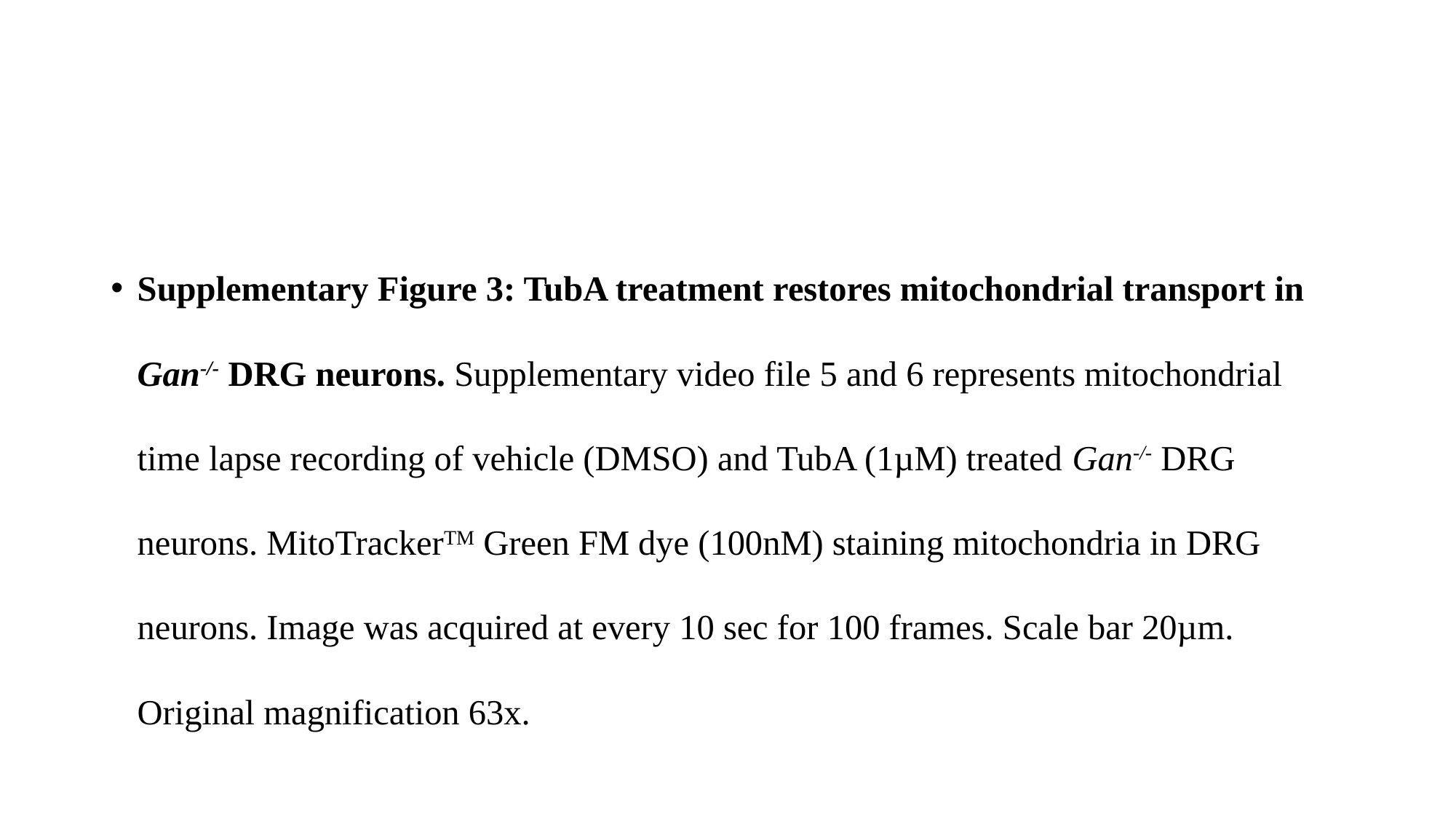

#
Supplementary Figure 3: TubA treatment restores mitochondrial transport in Gan-/- DRG neurons. Supplementary video file 5 and 6 represents mitochondrial time lapse recording of vehicle (DMSO) and TubA (1µM) treated Gan-/- DRG neurons. MitoTrackerTM Green FM dye (100nM) staining mitochondria in DRG neurons. Image was acquired at every 10 sec for 100 frames. Scale bar 20µm. Original magnification 63x.

## Slide 7
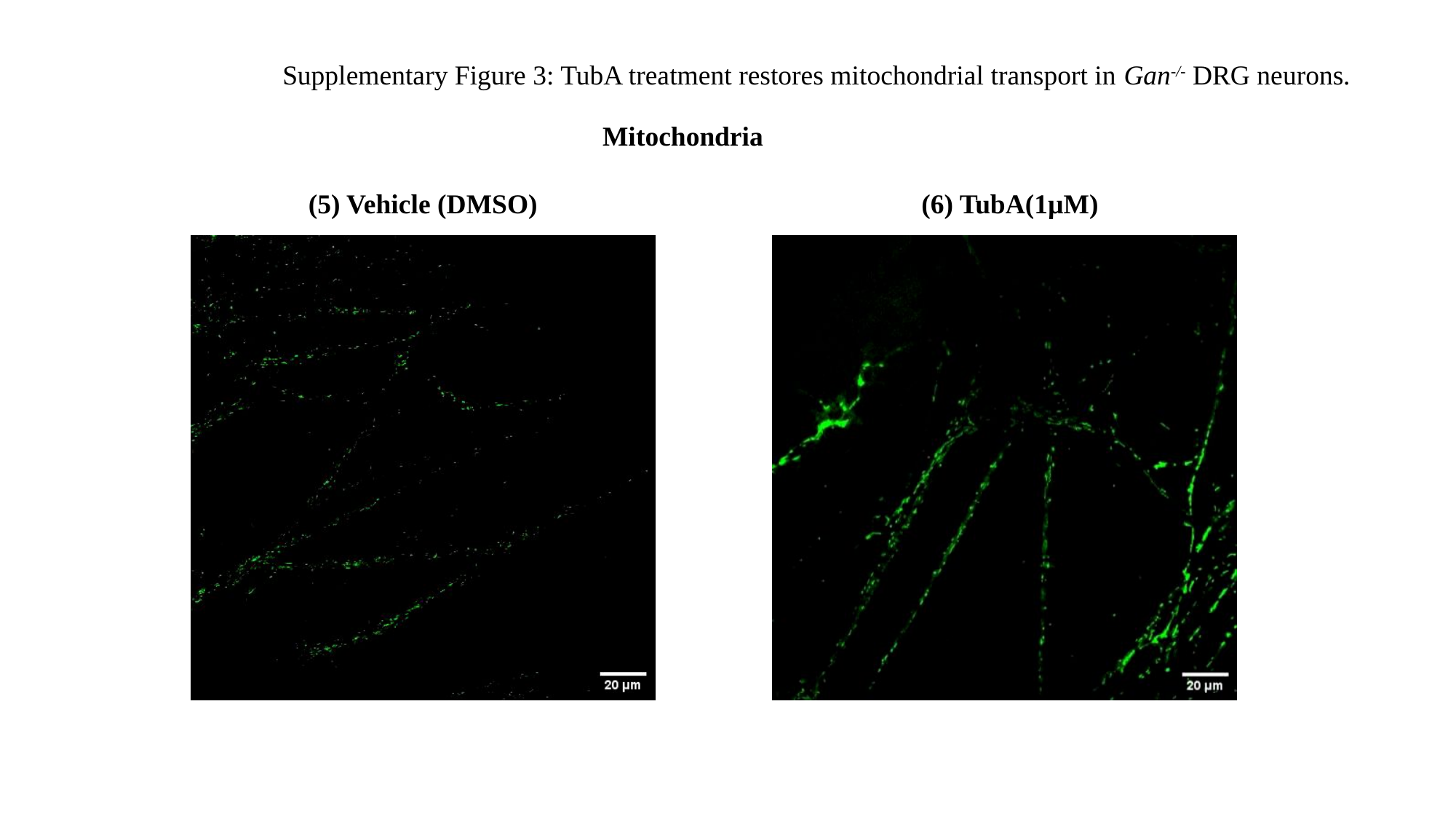

Supplementary Figure 3: TubA treatment restores mitochondrial transport in Gan-/- DRG neurons.
Mitochondria
(5) Vehicle (DMSO)
(6) TubA(1µM)

## Slide 8
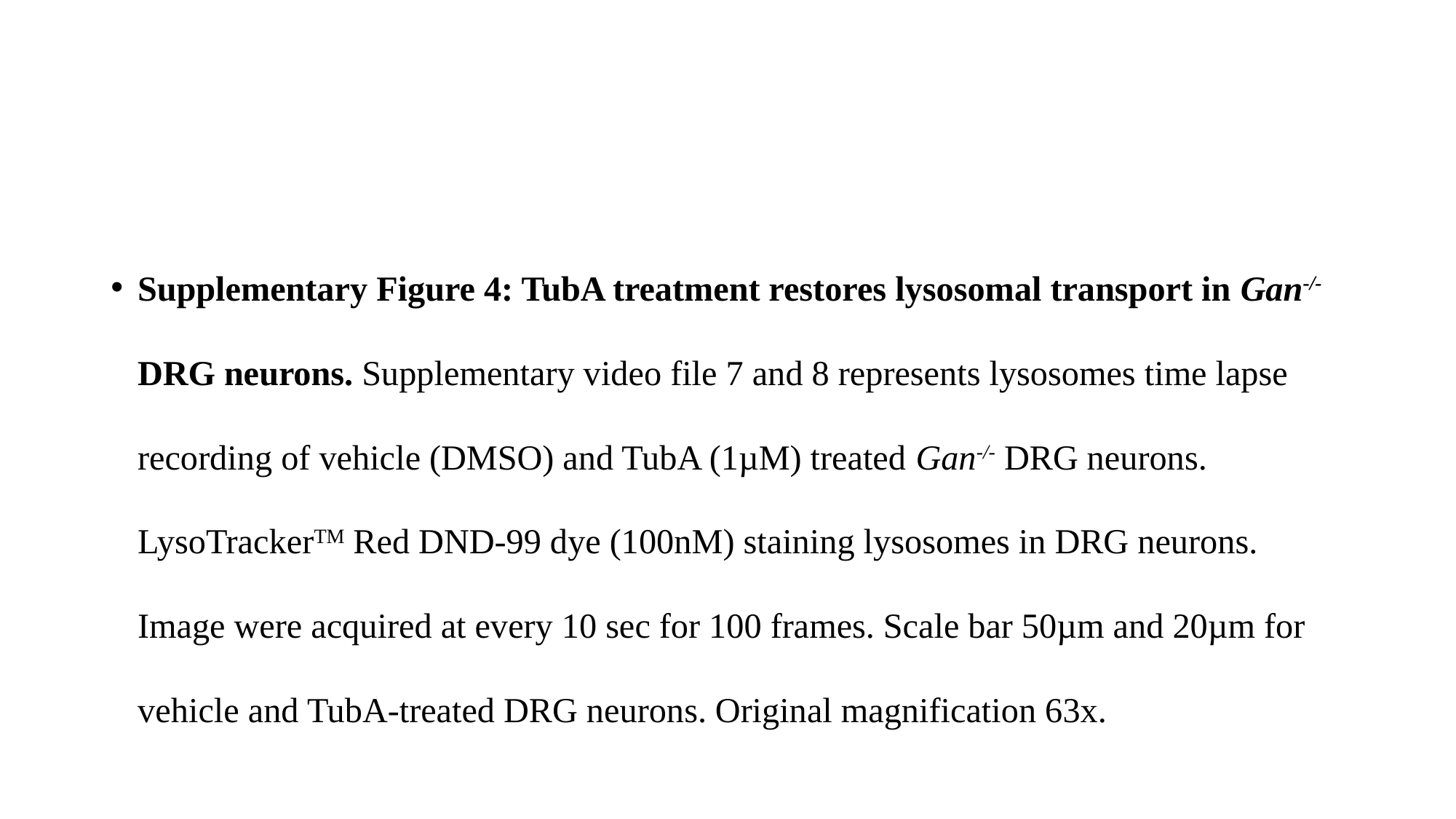

#
Supplementary Figure 4: TubA treatment restores lysosomal transport in Gan-/- DRG neurons. Supplementary video file 7 and 8 represents lysosomes time lapse recording of vehicle (DMSO) and TubA (1µM) treated Gan-/- DRG neurons. LysoTrackerTM Red DND-99 dye (100nM) staining lysosomes in DRG neurons. Image were acquired at every 10 sec for 100 frames. Scale bar 50µm and 20µm for vehicle and TubA-treated DRG neurons. Original magnification 63x.

## Slide 9
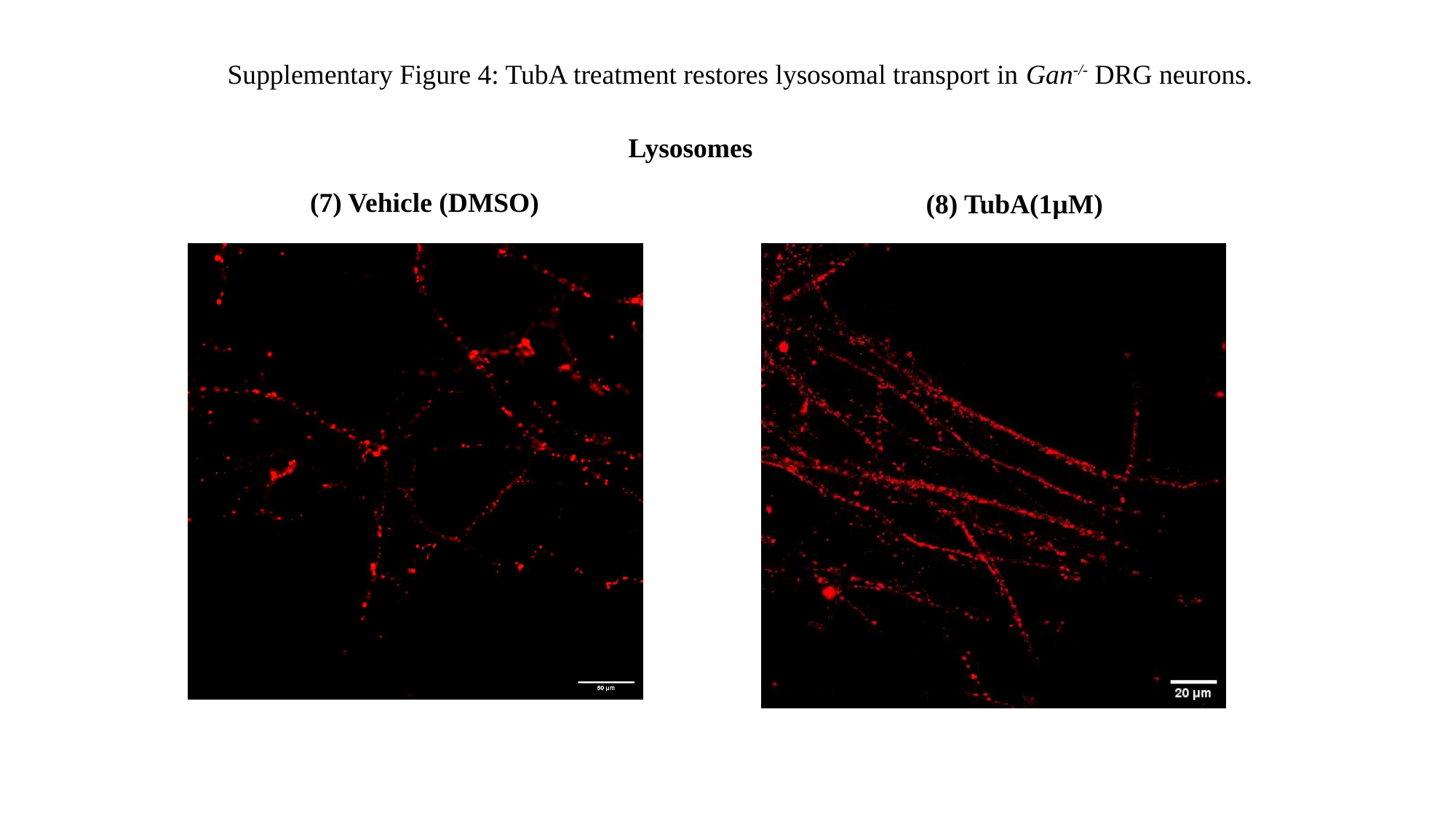

Supplementary Figure 4: TubA treatment restores lysosomal transport in Gan-/- DRG neurons.
Lysosomes
(7) Vehicle (DMSO)
(8) TubA(1µM)
